# 18 F-FDG-PET/MRI in patients with Graves’ orbitopathy.

**DOI:** 10.1007/s00417-021-05339-1

**Published:** 2021-08-18

**Authors:** Manuel Weber, Cornelius Deuschl, Nikolaos Bechrakis, Lale Umutlu, Gerald Antoch, Anja Eckstein, Ina Binse, Michael Oeverhaus

**Affiliations:** 1 Department of Nuclear Medicine, University Hospital Essen, Essen, Germany; 2 Institute for Diagnostic and Interventional Radiology and Neuroradiology, University Hospital Essen, Essen, Germany; 3 Department of Ophthalmology, University Hospital Essen, Hufelandstr. 55, 45147 Essen, Germany; 4 Department of Diagnostic and Interventional Radiology, Heinrich-Heine-University Düsseldorf, Düsseldorf, Germany

**Keywords:** Thyroid eye disease, GO, TED, PET, MRI, Imaging

## Abstract

**Purpose:**

Currently, therapeutic management of patients with Graves’ orbitopathy (GO) relies on clinical assessments and MRI. However, monitoring of inflammation remains difficult since external inflammatory signs do not necessarily represent the orbital disease activity. Therefore, we aimed to evaluate the diagnostic value of ^18^F-FDG-PET/MRI to assess the inflammation of GO patients.

**Methods:**

Enrolled patients with new onset of GO underwent ophthalmological examinations to evaluate the activity (CAS) and severity of GO (NOSPECS), as well as an ^18^F-FDG-PET/MRI (Siemens Biograph mMR) with dual time point imaging (immediately post-injection and 60 min p.i.). A subset of PET parameters including maximum standardized uptake value (SUVmax), metabolic target volume (MTV), and total lesion glycolysis (TLG) were obtained separately per eye and per extraocular eye muscle (EOM). EOM thickness was measured on the co-registered MRI.

**Results:**

Of 14 enrolled patients, three showed mild, seven moderate-to-severe, and four sight-threatening GO. Patients with severe GO showed statistically significant higher TLG than patients with mild GO (*p* = 0.02) and higher MTV than patients with mild (*p* = 0.03) and moderate (*p* = 0.04) GO. Correlation between NOSPECS on one hand and MTV and TLG on the other was significant (*R*^2^ = 0.49–0.61).

**Conclusion:**

TLG and MTV derived from FDG-PET appear to be good discriminators for severe vs. mild-to-moderate GO and show a significant correlation with NOSPECS. As expected, PET parameters of individual eye muscles were not correlated with associated eye motility, since fibrosis, and not inflammation, is mainly responsible for restricted motility. In conclusion, ^18^F-FDG-PET/MRI can be used for assessment of GO inflammation.

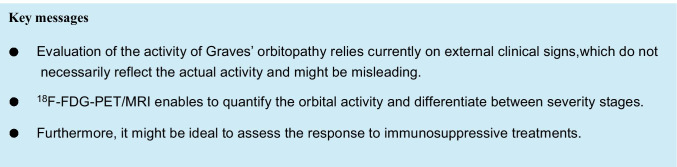

**Supplementary Information:**

The online version contains supplementary material available at 10.1007/s00417-021-05339-1.

## Introduction

Graves’ orbitopathy (GO), the most common extrathyroidal manifestation of Graves’ disease, is a disorder of autoimmune origin. Typically, patients show symptoms of inflammation of the orbital soft tissues, inflammatory triggered fibrosis of the ocular muscles, and adipogenesis [1, 2]. Consequently, patients suffer from signs of inflammation (pain, swelling), diplopia (due to fibrosis of extraocular muscles), and proptosis (due to adipogenesis), which has a serious impact on the quality of life of affected patients [3–6]. In the worst case, GO can cause vision loss through optic nerve compression [7]. Until now, there is no preventive therapy available for these patients, which is why current treatment mainly aims to temper inflammation. However, especially restrictive eye muscle changes persist with need of rehabilitative surgery afterwards [8–10]. Therefore, non-invasive diagnostic procedures that identify patients at risk for deterioration of GO are much needed in order to commence treatment before irreversible fibrosis and adipogenesis occur. Patients are classified as mild, moderate to severe, or sight threatening [8]. Mild GO can mostly be handled with selenium and supportive measures. However, some patients progress to moderate-to-severe GO and need to be identified early to start intravenous glucocorticoids (GCs) with or without orbital irradiation [8, 9, 11]. In the case of insufficient response, other immunosuppressive treatments, e.g., rituximab, tocilizumab, and mycophenolate mofetil, can be used. In some countries, teprotumumab, a new targeted therapy, is already approved and can be used [12]. Sight-threatening GO due to optic nerve compression or corneal exposure ulcers needs immediate treatment mostly with orbital decompression [13, 14]. However, these patients often do not show severe clinical inflammation and might be detected first with decreased visual acuity [15]. MRI allows for the high-resolution depiction of enlarged extraocular muscles and (using selected sequences, such as STIR, SIR, and DWI) enables the differentiation of patients with GO. The use of gadolinium-based contrast can provide additional information on the inflammation activity [16]. The identification of patients with active disease has important implications for treatment planning, as they need proper immunosuppressive treatment to prevent further progression with irreversible damage. Of note, clinical evaluation of inflammation activity entails a stronger predictor of treatment response than MRI parameters [17]. In recent years, a growing number of studies have suggested the immense potential of ^18^F-FDG-PET for imaging infection and inflammation, for example in the context of endocarditis or vascular prosthetic infections [18–21]. Therefore, ^18^F-FDG-PET/MRI could also serve as an early marker of increased inflammation and progression of GO before irreversible tissue changes occur. Prior studies have confirmed that individuals suffering from GO showed elevated FDG uptake in the extraocular eye muscles when compared to their non-affected counterparts [22]. If successful, GO patients could be treated properly before progressing to more advanced stages, thus reducing the need for rehabilitative surgery.

Therefore, the aim of this study was to assess whether ^18^F-FDG-PET is a valid diagnostic tool to assess the activity and severity of GO using clinical evaluation and magnetic resonance imaging as reference standard. ^18^F-FDG-PET was performed in conjunction with MRI (FDG-PET/MRI), because of the superior soft tissue contrast resolution of MRI as compared with CT. To evaluate the actual benefit of ^18^F-FDG-PET/MRI in GO management, we performed an interdisciplinary clinical trial in our tertiary GO referral center.

## Patients and methods

### Study population

For this clinical trial, we retrospectively analyzed patient records from patients who underwent a PET/MRI examination in our EUGOGO (European Group On Graves’ Orbitopathy) tertiary referral center between July 2017 and September 2018. All patients showed a new onset of GO and were referred to a PET/MRI due to difficult treatment decisions. Only patients with active GO (CAS ≥ 4) who were yet untreated, without prior surgeries, not pregnant or breast feeding were included in this clinical trial. The study was performed under adherence of the ethical foundations of the Declaration of Helsinki and was approved by the Ethics Commission of the University of Essen (11–4822-B0).

### Ophthalmological examination

Eye examinations were performed using a slightly modified EUGOGO case record form. First, all patients were evaluated by a highly trained orthoptist and afterwards by a specialized ophthalmologist (A.E.). GO was diagnosed in presence of typical clinical signs on ophthalmological examination, including slit-lamp biomicroscopy, applanation tonometry, funduscopy, Hertel and Naugel exophthalmometry, assessment of subjective diplopia and objective measurement of misalignment using the prism cover test, and measurement of monocular excursions and visual acuity. GO activity was evaluated using the CAS classification system established by Mourits et al. [23, 24]. By analysis of personal photos of the patients and anamnestic exploration concerning double vision and visual acuity, we determined the dynamic of the disease and scored CAS up to 10 points at baseline. GO was classified active with CAS values of ≥ 4/10 points (exception CAS 3/10, if recent onset of severe impairment of motility). Furthermore, we scored solely the soft tissue inflammation signs derived from CAS more gradually as follows: spontaneous retrobulbar pain (0–1), upper lid edema (0–2), lower lid edema (0–2), conjunctival injection (0–1), chemosis (0–1), lid redness (0–1), and swelling of caruncle or plica (0–1). The sum builds the clinical soft tissue score (STS) for each eye separately in comparison to the bilateral CAS.

In synopsis with MRI images, we classified the clinical severity of the patients according to the three EUGOGO grades: mild, moderate to severe, sight threatening. Additionally, scoring of the severity of GO (NOSPECS) was carried out according to the proposed criteria of the EUGOGO, as previously described [25, 26].

### Image acquisition

All scans (*n* = 14) were acquired on a Siemens Biograph mMR (Siemens Healthcare, Erlangen, Germany). Before the image acquisition, patients waited in a completely dark room. Additionally, they were instructed to keep their eyes closed prior to and during image acquisition to reduce functional ^18^F-FDG accumulation in the extraocular muscles (EOM). A mean (± standard deviation) of 227 (± 40) megabecquerel FDG was administered on the PET imaging table and scan acquisition including an early (immediately p.i.) and late (60 min p.i.) static scan started immediately. Image reconstruction was performed in 3D mode using ordinary Poisson ordered subsets expectation maximization (3 subsets, 21 iterations) and a 4-mm Gaussian filter for post-smoothing, as published previously [27].

### Static PET analysis

PET analysis was carried out on a per-eye basis and separately for each EOM both for the early and late static images using co-registered MRI for anatomical orientation.

Per-eye analysis (PEA) was executed using SyngoVia (Siemens Healthcare, Erlangen, Germany) by placing a spheric region of interest (ROI) in the orbital structures.

Additionally, individual extraocular muscle segmentation (IEMS) was performed for all rectus muscles with a 2D brush tool using MIM version 6.9.5 (MIM Software Inc., Cleveland, OH).

For both analyses, a 70% threshold was employed to exclude tissue without relevant tracer accumulation from further evaluation.

Subsequently, mean and maximum standardized uptake values (SUVmean, SUVmax) and metabolic target volume (MTV) and total lesion glycolysis (TLG) were extracted from the established ROIs. MTV was defined as the volume of the orbital tissue with an uptake of ≥ 70% of local SUVmax; TLG was defined as the product of MTV and SUVmean of the respective ROI. Furthermore, the presence of increased uptake in paranasal sinuses was recorded on a binary yes/no scale.

For the MRI analysis, the maximum horizontal diameters of the inferior, superior, and medial rectus muscles were measured by a blinded neuroradiologist, as well as the activity in terms of contrast enhancement (CD).

### Statistical evaluation

For metric data, median values and range or the mean and standard deviation (SD ±) were calculated and differences between groups were evaluated with Student’s *t*-test (two-tailed) if the D’Agostino-Pearson omnibus normality test showed normal distribution, if not with Mann–Whitney test. Fisher’s exact test was used to evaluate group distributions of binary variables. Kruskal–Wallis was employed to analyze differences of PET parameters among GO classes. Linear regressions have been performed to test the association between clinical and PET/MRI parameters. Multiple linear regression has been performed to assess further influential factors. Level of statistical significance was defined two-tailed as 2*α* < 0.05. All calculations were performed with SPSS (IBM SPSS Statistics, Chicago, IL, USA, version 22.0.0) and GraphPad Prism (Prism 8 for Windows, Software Inc., San Diego, CA, USA, version 8.01). *p*-values are given with α-adjustment for multiple testing.

## Results

### Study population

Fourteen GO patients were enrolled and analyzed. Three showed mild, seven moderate-to-severe, and four sight-threatening GO. The patient cohort consisted of 11 women (79%) and 3 men (21%), and the median age at imaging was 49.9 years (range: 30–71). An overview of patients’ characteristics is provided in Table 1.

**Table 1:** Baseline characteristics of the study population

	*n*	
Age (years)	14	49.9 ± 11 [30–71]
Females	11	78.6%
Smoker	11	78.6%
Cigarettes per day	11	17 [8-35]
Previous antithyroid treatments		
Methimazole	11	78.6%
Radioiodine therapy	4	28.6%
Thyroidectomy	4	28.6%
Duration of GO (months)	14	7.5 ± 5
EUGOGO stage		
Mild	3	21.4%
Moderate to severe	7	50%
Sight threatening	4	28.6%
CAS	14	5.0 ± 1
NOSPECS	14	5.7 ± 3
Protrusion preoperatively (in mm)	28	18.7 ± 3.7
Diplopia	7	50%

Unless otherwise stated, data are means ± SD or proportions (%) or median [range]

*CAS*, clinical activity score; *NOSPECS*, clinical severity score; *PD*, prism diopters; *BSV*, binocular single vision

### Correlation between PET parameters and inflammation

**Fig. 1:**
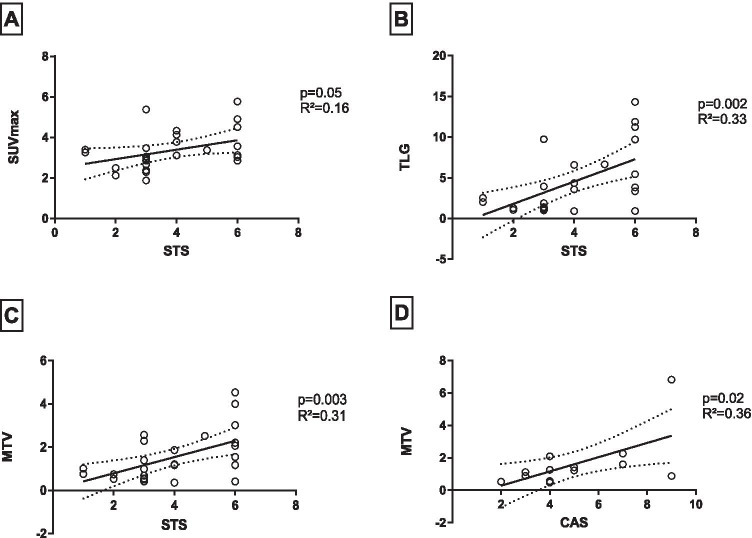
Linear regression showed a significant correlation between clinical activity (soft tissue inflammation score, STS) and PET parameter SUVmax (**A**), total lesion glycolysis (**B**), metabolic target volume (**C**) per eye, as well as for bilateral (**D**) clinical activity (CAS, clinical activity score) and bilateral PET parameter (metabolic target volume)

To analyze the correlation between clinical signs of inflammation and the activity measured with ^18^F-FDG PET (early acquisition), we performed linear regressions. We found a statistically significant correlation between MTV (*p* = 0.003, *R*^2^ = 0.31), TLG (*p* = 0.002, *R*^2^ = 0.33), and SUVmax (*p* = 0.05, *R*^2^ = 0.16) on the one hand, and the soft tissue inflammation score (STS) on the other hand (Fig. 1). Each eye was evaluated separately.

Furthermore, we observed statistically significant correlations with the CAS for the following bilateral PET parameters derived from the late static images: TLG (*p* = 0.04, *R*^2^ = 0.31) and MTV (*R*^2^ = 0.36; *p* = 0.02, Fig. 1D). In contrast, PET parameters from the early static images showed no significant correlation with CAS.

Whereas 3 of 4 sight-threatening and 2 of 7 moderate-to-severe patients showed increased uptake in paranasal sinuses, this was not present in any mild GO. Bilateral TLG was significantly higher in patients with increased uptake in paranasal sinuses compared to those without (10.9 vs. 4.5, *p* = 0.04).

Furthermore, we investigated the correlation between MRI and PET. Patients with contrast enhancement showed a borderline statistically higher bilateral TLG (*p* = 0.05) compared to patients without it. MTV showed only a statistical trend (*p* = 0.07). Clinical activity parameter (STS, CAS) showed no significant differences (*p* > 0.46).

### Differences in PET parameters among severity-stratified groups

**Fig. 2:**
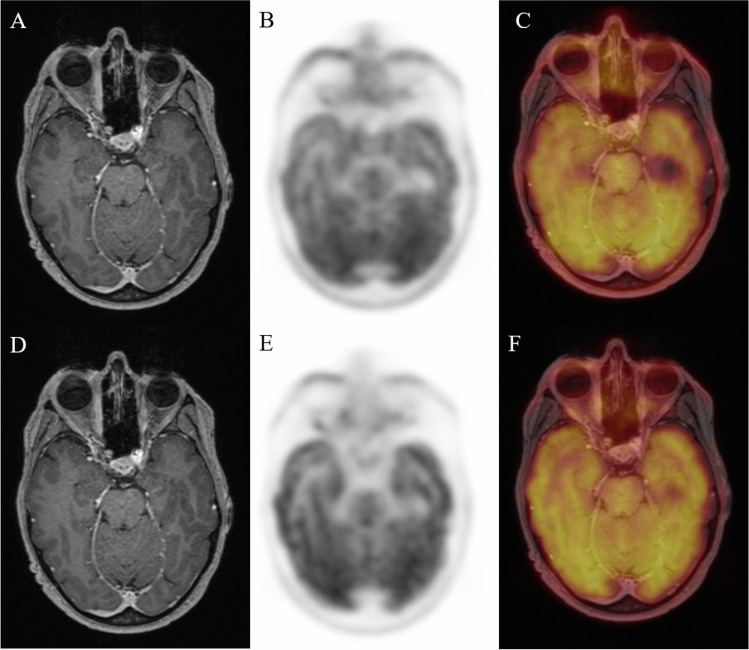
Transaxial MRI (panels **A**, **D**), PET (panels **B**, **E**), and fused slices of a 39-year-old patient with mild GO reveal slight hypertrophy of orbital tissue and faint ^18^F-FDG uptake, both on early (panels **A**–**C**) and late static images (panels **D**–**F**)

**Fig. 3:**
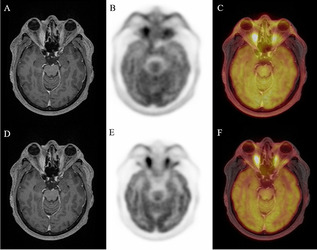
Transaxial MRI (panels **A**, **D**), PET (panels **B**, **E**), and fused slices of a 34-year-old patient with severe GO reveal pronounced orbital tissue hypertrophy, corresponding with high and extensive bilateral ^18^F-FDG uptake, both on early (panels **A**–**C**) and late static images (panels **D**–**F**)

**Fig. 4:**
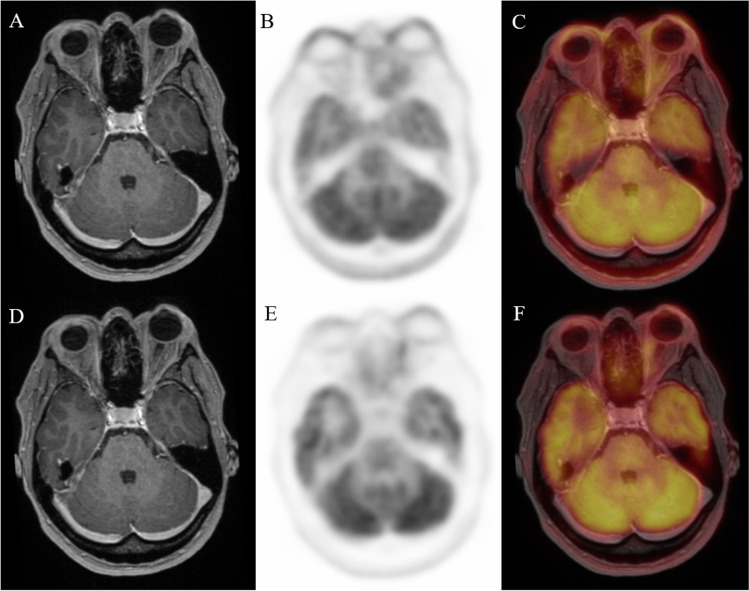
Transaxial MRI (panels **A**, **D**), PET (panels **B**, **E**), and fused slices of a 21-year-old patient with moderate, mainly left-sided GO reveal orbital tissue hypertrophy and ^18^F-FDG uptake, both more pronounced on the left side. The latter is more distinct on early static (panels **A**–**C**) vs. late static images (panels **D**–**F**)

To explore the capability of ^18^F-FDG PET to differentiate the severity of GO patients, we compared PET parameter between the three EUGOGO severity grades (mild, moderate to severe, and sight threatening). MRI and PET images of patients with mild, severe, and unilateral GO are provided in Figs. 2, 3, and 4.

**Fig. 5:**
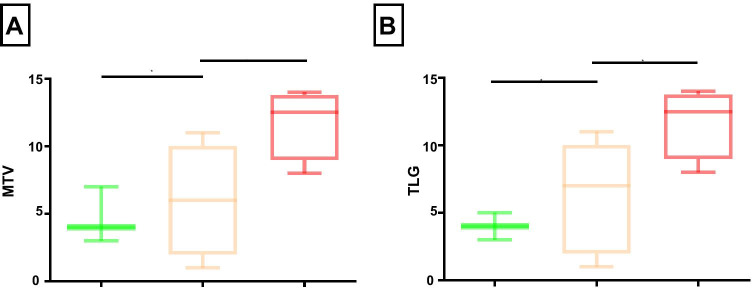
Mild (green) and moderate-to-severe (orange) afflicted patients showed significantly lower bilateral MTV and TLG values compared with sight-threatening cases (red). *p < 0.05; MTV, metabolic target volume; TLG, total lesion glycolysis

Bilateral TLG and MTV values showed significant differences between mild/moderate-to-severe and sight-threatening cases (Fig. 5). However, differences in TLG (4.0 vs. 6.7, *p* = 0.35) and MTV (4.7 vs. 6.3, *p* = 0.58) were not statistically significant between patients with mild vs. moderate GO. Summed bilateral TLG obtained from the early static images was significantly higher in patients with sight-threatening GO when compared to patients with mild (median 19.7 vs. 3.7; *p* = 0.02) GO. Differences to patients with moderate GO were less but still statistically significant (median 19.7 vs. 4.8; *p* = 0.05). Summed MTV derived from the early static images was significantly higher in patients with sight-threatening GO when compared to patients with mild (median 5.7 vs. 1.4; *p* = 0.03) or moderate (median 5.7 vs. 1.7; *p* = 0.04) GO.

Summed MTV obtained from late static images was higher in patients with sight-threatening vs. moderate (9.8 vs. 7.4, *p* = 1.0) GO or vs. mild GO (9.4 vs. 4.7, *p* = 0.33), and higher in moderate vs. mild GO (7.4 vs. 4.7, *p* = 1.0), but statistical significance was not reached. Similarly, summed TLG did not differ statistically significantly among groups (mild vs. moderate: 5 vs. 7.3, *p* = 1.0; moderate vs. severe: 7.3 vs. 9.8, *p* = 1.0; mild vs. severe: 5 vs. 9.8, *p* = 0.41).

**Fig. 6:**
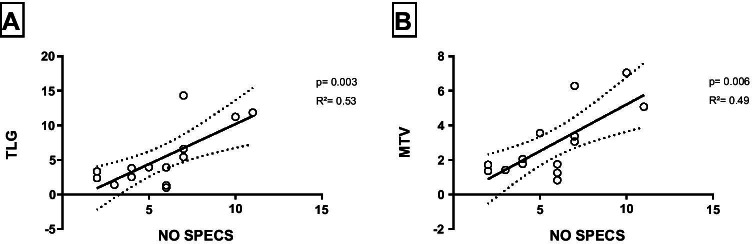
Summed bilateral TLG and MTV showed a significant correlation to the clinical severity assessment score (NOSPECS). MTV, metabolic target volume; TLG, total lesion glycolysis

For further exploration of the correlation between clinical severity assessment and PET parameter, we performed linear regressions: On the early static images, a statistically significant correlation between NOSPECS and TLG (summed TLG: *R*^2^ = 0.53, *p* = 0.003) and between NOSPECS and MTV (summed MTV: *R*^2^ = 0.49, *p* = 0.006) was observed. Similarly, a statistically significant correlation between TLG (summed TLG: *R*^2^ = 0.61, *p* = 0.001) and MTV was observed (summed MTV: *R*^2^ = 0.52, *p* = 0.004) on the late static images (Fig. 6).

### Detailed muscle analysis

**Fig. 7:**
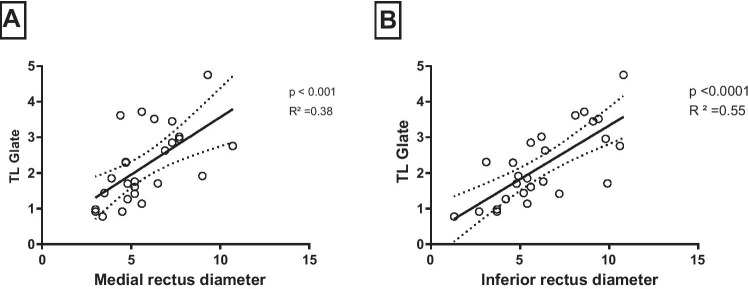
The detailed single rectus muscle analysis showed that the clinically most often afflicted eye muscles show a significant correlation between muscle diameter in MRI and TLG, more prominently in the late static images. TLG, total lesion glycolysis

To further explore the muscle inflammation, each rectus muscle was evaluated separately for each eye in PET and MRI images. We observed a statistically significant correlation between the rectus medial muscle diameter on the one hand and SUVmax, and TLG derived from the early static images on the other hand (*R*^2^ = 0.25–0.26, *p* < 0.01). In late static images, only TLG showed a significant correlation (*R*^2^ = 0.38, *p* < 0.001, Fig. 7). The diameter of the inferior rectus muscle was also significantly correlated with SUVmax, SUVmean, MTV, and TLG derived from the early static images (*R*^2^ = 0.29–0.41, *p* > 0.001). Similarly, late static images showed only a significant correlation with TLG (*R*^2^ = 0.55, *p* < 0.0001). No statistically significant correlation was observed between the superior and lateral rectus muscles and PET parameters in early and late static images.

As assessed by Pearson’s correlation coefficient, no significant correlations between the PET parameters obtained from IEMS on the one hand, and motility of the inferior (*p* = 0.21–0.95), lateral (*p* = 0.29–0.75), medial (*p* = 0.07–1.0), and superior (*p* = 0.20–0.67) rectus muscles were observed.

Regarding the severity and eye muscle involvement, group comparison showed that patients with sight-threatening GO had significant higher TLG compared to mild and moderate to severe (median 30.9 vs. 8.3/9.9, *p* = 0.03). Mild and moderate-to-severe patients showed similar muscle activity in PET.

## Discussion

In this retrospective study, we were able to show that ^18^F-FDG-PET/MRI is a tool to assess the activity of patients with Graves’ orbitopathy. In synopsis with the clinical assessment, it might be beneficial for the distinction of mild vs. moderate-to-severe vs. sight-threatening GO and identification of patients who need more aggressive treatments. However, the PET/MRI is a lengthy examination and needs a very precise setting to minimize confounding of the examination by eye movements. Furthermore, PET/MRI availability is currently limited. Technical advances to achieve shorter protocols and new tracer that are less sensitive to confounding might enable the use of PET/MRI as a diagnostic tool in GO for the clinical routine in the future. Currently, it can be used as an academic tool to elucidate the pathophysiology and course of GO.

### Correlation between PET parameters and inflammation

An important goal when treating GO patients is to assess the activity of the disease to initiate adequate anti-inflammatory treatments and start rehabilitative surgery in the inactive phase. Before treatment MRI provides the opportunity by evaluation of contrast enhancement, STIR and DWI sequences to assess the activity of the disease. However, it is known that the visible changes persist even in inactive patients, which is why MRI does not allow to control the treatment response [17]. ^18^F-FDG-PET measures the glucose metabolism and is therefore theoretically an ideal tool to measure inflammation, because of the increased metabolism. The usefulness has been shown for several other autoimmune and infectious diseases, e.g., Crohn’s disease and rheumatoid arthritis [18, 28–31]. This is why we used PET/MRI examinations in patients with new onset of GO and special circumstances (comorbidities, severely decreased quality of life) to determine the initial activity of the disease and monitor the response to the treatment. Our cohort analysis showed that the soft tissue inflammation score (STS) per eye, CAS, and MRI contrast enhancement correlated well with the activity measured by PET. Patients who showed activity in MRI images showed also higher TLG values. Therefore, ^18^F-FDG-PET might be a useful addition to assess the activity in GO patients, especially to monitor the response during anti-inflammatory treatments. This is of special importance since non-responders to steroid treatment need to be quickly identified to initiate different immunosuppressive drugs (e.g., tocilizumab, MMF) to prevent further irreversible damage. Furthermore, it could be used to identify patients with high inflammation for their disease stage on the brink of progression of the disease severity. Then, therapy could be augmented to prevent irreversible damages.

To further elucidate the role of the microbiome in GO, we analyzed the presence of increased FDG uptake in paranasal sinuses [32]. Our analysis showed more often high activity sinuses in more severely afflicted GO patients which might indicate that the paranasal microbiome has a role in the commonly known heterogeneity of the disease. Naturally, this finding is limited by the small case number and should be subject to further studies.

### Differences in PET parameters among severity-stratified groups

Since the clinical grading of patients into mild, moderate-to-severe, and sight-threatening cases is not always easy and mandatory for individual and adequate treatment, we used ^18^F-FDG-PET as an additional diagnostic tool. While conventional PET parameters such as SUVmax or SUVmean fail to stratify EO patients according to EUGOGO grades, TLG and MTV appear to have good discriminatory power: Patients with severe GO had higher per-eye MTV and TLG on the early static images; however, no statistically significant differences were shown on the late static images. In contrast, TLG and MTV obtained both from early and late static images showed a good correlation with NOSPECS scores, the severity score in GO. However, this might be explained since inflammation is one of the metrics for NOSPECS assessment in contrast to the EUGOGO severity grading. In general, we would expect no correlation between PET parameter and severity in inactive patients. In our cohort, we examined only newly diagnosed active GO patients which is probably why the inflammation in PET is significantly different between severity grades.

The lack of correlation between SUVmax and severity of GO is in line with prior publications on this topic: García-Rojas et al. analyzed the ^18^F-FDG-PET/CT scans of 16 patients with GO and found that FDG uptake in the EOM as assessed by SUVmax was significantly higher than in the non-affected control group [22]. However, a subsequent study by the same group did not find any correlation between SUVmax on the one hand and muscle thickness and clinical inflammation score on the other hand [33]. Similarly, a study by Uslu-Beşli et al. showed that ^18^F-FDG uptake as assessed by SUVmax was elevated in patients with GO when compared to a non-affected control group but they as well did not find a correlation between SUVmax and muscle thickness [34]. Contrarily to our protocol, in these studies, dual time point PET was not performed, and PET analysis only comprised the measurement of SUVmax. Finally, PET scans in our study were acquired on a hybrid PET/MRI with simultaneous MRI acquisition that provides more anatomical information due to the better soft tissue contrast when compared to a CT scan. Our results in conclusion with the prior publications on PET in GO might indicate that SUVmax is not an ideal parameter to assess GO. This might be due to the fact that in contrast to TLG and MTV, it only measures the maximum uptake and does not take the volume of the inflammatory tissue into account.

The better discriminatory power of early static PET images constitutes an interesting finding, as the current EANM/SNMMI Guideline for ^18^F-FDG Use in Inflammation and Infection recommends an uptake interval of ≥ 60 min [35]. Prior studies on tumor patients have shown early ^18^F-FDG uptake to be correlated with perfusion [36]. It might therefore also be reflective of the bigger size of the EOM in this group, which would lead to a higher volume of activity distribution. Still, the inferior and medial rectus diameter showed a moderate correlation with TLG (*R*^2^ = 0.38/0.55) indicating that there are more influential factors present. While the muscle size reflects mainly the edema caused by inflammation, FDG uptake reflects the increased metabolic activity due to inflammatory responses. Therefore, ^18^F-FDG-PET might be especially beneficial for early detection of increased inflammation before edema and later fibrosis leads to an increased muscle size as well as treatment monitoring since muscle fibrosis does not resolve as fast as activity. Thus, ^18^F-FDG-PET/MRI might provide the imaging correlate of the inflammatory component in GO and thus overcome current limitations of MRI.

In the detailed muscle analysis, we observed a lack of correlation between PET parameters on the one hand and EOM motility on the other hand. As the main factor for limited EOM motility in GO is fibrosis and not inflammation, this finding is not unexpected.

### Limitations

One limitation of our study is that due to the retrospective nature of this study, only 3 patients with mild GO were examined, which is a possible explanation for the lack of statistically significant differences in PET parameters when comparing patients with mild vs. moderate GO. Furthermore, we cannot preclude that the PET measurements were confounded by eye movements, although we followed a strict protocol in a dark room with adaption period and examined only suitable patients. Still, the consistency of the measurements indicates that mainly inflammation and not muscle activity was measured. Further studies on larger collectives in a prospective manner should be performed to verify the results from this pioneer study. These studies should also include patients with clinically inactive GO to assess the use of PET-MRI in this stage of the disease.

Future trials might also elucidate the potential of PET imaging using fibroblast activation protein inhibitors (FAPI) in patients with GO: Since fibrosis plays a pivotal role in the pathogenesis of GO, the portrayal of fibroblast activation might allow for an accurate assessment of disease activity without being susceptible to eye movement as FDG.

## Conclusion

^18^F-FDG-PET/MRI might be a useful method to precisely assess the inflammation of different tissues in GO. Due to the complex nature and rare availability, it is currently not recommendable for clinical routine. In special cases, it might be beneficial to accurately assess and monitor the inflammation which might be challenging with current methods. However, further studies with larger collectives and follow-up data are needed.

## Supplementary Information

Below is the link to the electronic supplementary material.

Supplementary file1 (DOCX 92 kb)

## Data Availability

The datasets generated during and/or analyzed during the current study are available from the corresponding author on reasonable request.

## Code availability

Not applicable for this manuscript.
