# Supplementary material for: 18 F-FDG-PET/MRI in patients with Graves’ orbitopathy
Source: Graefes Arch Clin Exp Ophthalmol. 2021 Aug 18;259(10):3107–17. doi: 10.1007/s00417-021-05339-1 (PMC8478760; doi:10.1007/s00417-021-05339-1)
Supplement: Supplementary file 1 — Supplementary file1 (DOCX 92 kb) [file 417_2021_5339_MOESM1_ESM.docx]

**Supplemental figure 1**: Linear regression showed a significant correlation between clinical activity (CAS) and bilateral PET parameter Total lesion glycolysis
